# A pair of promising immune checkpoints PSGL-1 and VISTA from immunotolerance to immunotherapy

**DOI:** 10.1186/s40364-024-00693-8

**Published:** 2024-12-02

**Authors:** Manqing Peng, Xiaofang Lu, Junshuang Guo, Xiangli Yin, Jing Zhang, Xin Li, Yizhou Zou

**Affiliations:** Department of Immunology, School of Basic Medicine, Central South University, Changsha, Hunan 410000 China

**Keywords:** VISTA, PSGL-1, Cancer, Autoimmune diseases, Immune tolerance, Immunotherapy

## Abstract

Immune checkpoints are crucial for regulating immune responses and maintaining self-tolerance, as they play a pivotal role in preventing autoimmunity and facilitating tumor immune evasion. This review concentrates on the immune checkpoint molecules PSGL-1 and VISTA. Both molecules are highly expressed in hematopoietic cells, including T cells and myeloid cells. VISTA functions both as a ligand on myeloid cells, where it regulates cytokine production, chemotaxis, and phagocytosis while promoting their differentiation into a tolerogenic phenotype and as a receptor on T cells, where it contributes to T cell quiescence. PSGL-1, which acts as a binding partner for VISTA, further inhibits T-cell activation and fosters tolerance within the acidic tumor microenvironment. Our review provides a comprehensive analysis of the structure, expression, and biological functions of PSGL-1 and VISTA and emphasizes their therapeutic potential in cancer treatment, autoimmune diseases, and transplantation. The dual role of these checkpoints in immune regulation presents novel opportunities for advancing cancer immunotherapy and developing new strategies for managing autoimmune conditions.

## Introduction

Immune checkpoints are critical molecules on the surface of immune cells that exert negative immune regulation, mediating self-tolerance, preventing autoimmunity, and facilitating tumor immune escape [1]. With the in-depth study of the immune checkpoint mechanism, treatment targeting CTLA-4, PD-1/PD-L1, and other immune checkpoints has demonstrated efficacy in the treatment of a range of malignancies, including urothelial carcinoma(UC) [2], renal cell carcinoma(RCC) [3], melanoma [4], non-small-cell lung cancer(NSCLC) [5], colorectal carcinoma(CRC) [6] and Hodgkin’s lymphoma(HL) [7]. Despite these advancements, immune-related adverse events (IrAEs) such as autoimmune reactions, drug resistance, and delayed responses continue to pose challenges [8–10]. This underscores the need to explore second-generation immune checkpoint molecules.

P-selectin glycoprotein ligand-1 (PSGL-1), also known as CD162, is a 120 KDa type I transmembrane glycoprotein that typically exists as a 240 KDa homodimer. It is encoded by the SELPG gene situated on chromosome 12q24.11. PSGL-1 interacts with several ligands, including selectins P, E and L [11–13], CCL19 and CCL21 [14], Versican [15], Siglec-5 [16], amidase LytA [17] and VISTA [18]. PSGL-1 has long been studied as an adhesion molecule involved in immune cell migration [19, 20]. However, recent researchers suggest that PSGL-1 also functions as a negative regulator of the immune system [21, 22]. Notably, the interaction between PSGL-1 and VISTA has been shown to negatively regulate immune cell functions, particularly T cells [18].

V-domain immunoglobulin T-cell activation suppressor (VISTA), also known as PD-1 H [23], c10orf54 [24], DD1α [25], Gi24, and Dies1 [26], is a 34 KDa type I transmembrane protein encoded by the VISR gene on chromosome 10q22.1. VISTA belongs to one of the members of the B7 family of negative immune checkpoints. The most studied ligands of VISTA include VSIG-3 [27]and PSGL-1 [18], while several potential ligands have recently been identified, such as galectin-9 [28], VSIG-8 [29], MMP-13 [30], Sdc-2 [31]and LRIG-1 [32]. VISTA functions both as a ligand and receptor [33]. It can not only indirectly inhibit T cell activation by inducing myeloid cell differentiation towards a tolerogenic phenotype but also directly affect T cells by inhibiting their proliferation and activation, and inducing Foxp3^+^ expression to exert immunosuppressive effects [34, 35]. These mechanisms are crucial in the context of autoimmune diseases [36], graft-versus-host disease(GVHD) [24] and tumors [35].

In summary, we particularly focus on the pair of PSGL-1 and VISTA second-generation immune checkpoint molecules. this review emphasizes the structure, expression, interactions, and biological effects of PSGL-1 and VISTA. We will also discuss recent research advancements concerning their roles in immune tolerance, tumor escape, and clinical treatment. Our goal is to highlight these molecules as potential targets for novel cancer therapies and to explore their applications in autoimmunity and transplantation.

## The structure and interaction between PSGL − 1 and VISTA

PSGL-1 is a glycoprotein composed of 412 amino acids, organized into three structural domains: the extracellular domain (318 amino acids), the transmembrane domain (24 amino acids), and the cytoplasmic domain (70 amino acids) [37]. Glycosylation of the N-terminal end of the extracellular structural domain is essential for binding to P- [38], E- [39], and L-selectins [13]. Additionally, the extracellular domain of mature PSGL-1 is rich in threonine, proline, and serine, contributing to its decameric glycoprotein backbone, with human polymorphism in the tandem decameric repeat sequence [39]. The transmembrane domain contains cysteine residues that form disulfide bonds, facilitating dimerization. The cytoplasmic domain primarily interacts with ERM proteins (ezrin/radixin/moesin), which support PSGL-1-induced signaling [12, 40]. The transmembrane and cytoplasmic structural domains exhibit high conservation [37]. Thus, although the extracellular structural domain of PSGL-1 varies among species, its overall structure and function appear to be similarly regulated.

VISTA is a transmembrane protein composed of 279 amino acids, with features of four structural domains: the extracellular immunoglobulin IgV-like domain (130 amino acids), the stalk (33 amino acids), the transmembrane structural domain (20 amino acids) and the cytoplasmic tail (96 amino acids) [41, 42]. An analysis of the VISTA structure revealed that the extracellular structural domain is similar to that of the B7 family member PD-L1, with a notably larger IgV-like domain but lacking an IgC-like domain. The extracellular domain adopts a typical β-sandwich structure and features a significant number of positively charged histidine residues at the C-C’ loop. These residues contribute to the formation of pH-dependent binding sites, which facilitate VISTA’s interaction with ligands in acidic environments [43]. The cytoplasmic portion resembles CD28 family members PD-1 and CTLA-4, but it notably lacks the ITIM, ITAM, and ITSM motifs. The cytoplasmic portion of VISTA has a conserved Src homology 2 (SH2) binding motif (YxxQ) and three C-terminal SH3-binding domains (PxxP) as well as the phosphokinase C (PKC) and specific sequences in the casein kinase 2 (CK2) binding site [43, 44], which can alter certain functions within the cell and allow VISTA to act as both a ligand and a receptor [33]. Given its sequence similarity to other members of the B7 family, particularly in the extracellular domain, VISTA is classified as a B7 member. VISTA is the most conserved member of the B7 family, with 76% homology between mouse and human, and 90.6% identity between the cytoplasmic tails of human and mouse, which suggests that its functional role is highly conserved [42].

**Fig. 1:**
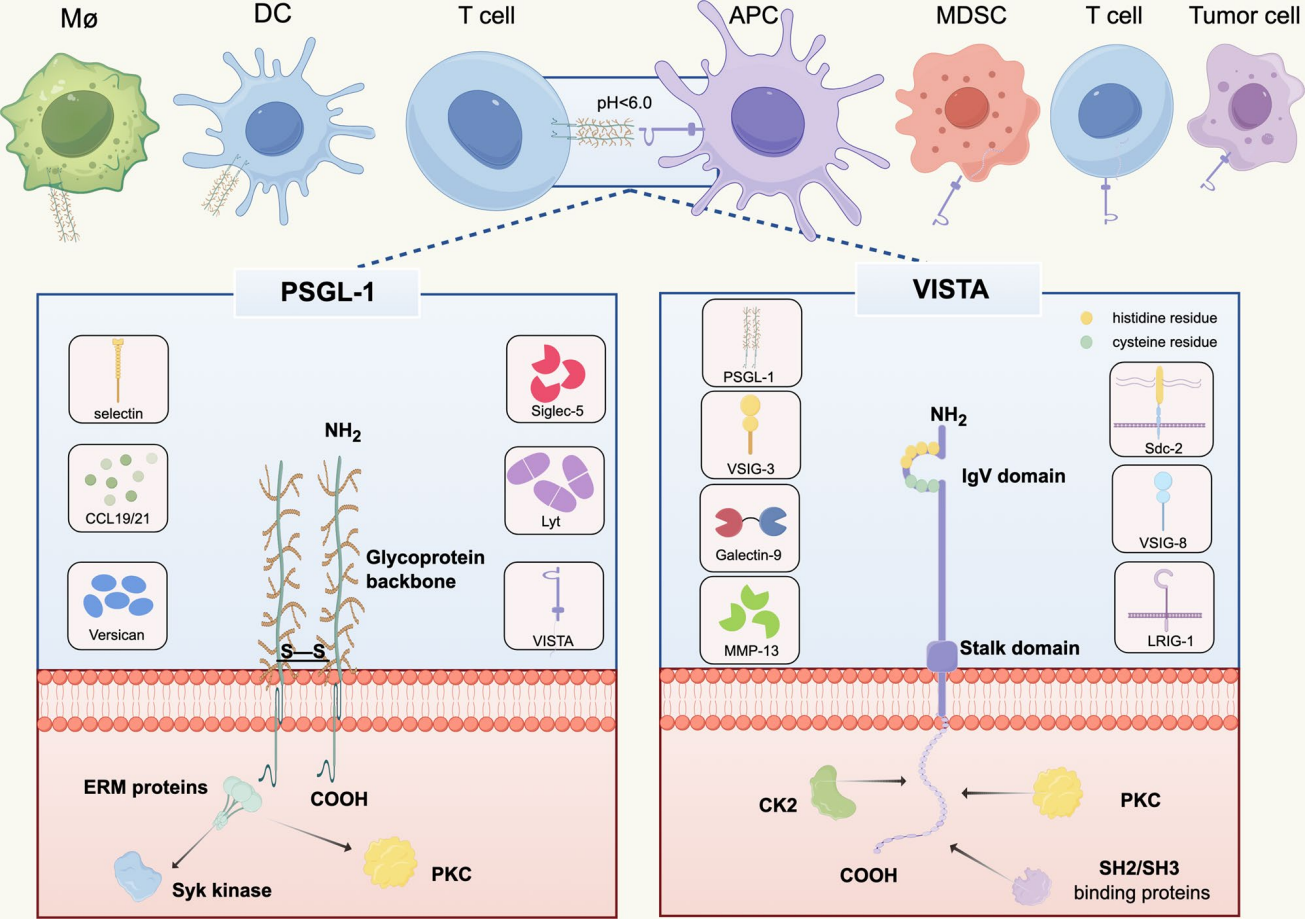
The structure and interaction between PSGL-1 and VISTA. PSGL-1 and VISTA interact with each other to exert inhibitory functions, particularly under acidic conditions. PSGL-1 is a dimeric protein with an extracellular region characterized by a decameric glycoprotein backbone. The transmembrane domain contains cysteine residues that form disulfide bonds, facilitating dimer formation. The cytoplasmic domain primarily interacts with ERM (ezrin, radixin, moesin) proteins to support PSGL-1-induced signaling. VISTA is a transmembrane protein that contains an IgV-like domain with an extended C-C’ loop and exceptional clusters of histidine which facilitate VISTA’s interaction with its ligands in an acidic environment. The cytoplasm conserves SH2 and three SH3 binding domains as well as binding sites for protein kinase C (PKC) and casein kinase 2 (CK2), which can alter certain functions within the cell and allow VISTA to act both as a ligand and a receptor. (By Figdraw)

The specificity and pH selectivity of PSGL-1 and VISTA binding was recently demonstrated in a study by Johnston et al. [18], where the researchers modeled the molecular docking of PSGL-1 with VISTA, which showed that sulfated tyrosine residues Y46 and Y48 of PSGL-1 ionically interact with protonated histidine residues H153 and H154 of VISTA. Furthermore, VISTA histidine residues H98 and H100 appear to interact with PSGL-1 residues E56 and Y51. For PSGL-1, the binding of PSGL-1 to VISTA is dependent on tyrosine sulfation. In VISTA, histidine residues H153, H154, and H155 have been demonstrated to facilitate binding to PSGL-1. In other words, the abundance of histidine residues in the extracellular structural domain of VISTA allows for pH-selective interactions with its ligand, and this inhibition occurs in acidic environments. This indicates that VISTA and PSGL-1 mediated immune responses may be regulated by pH-specific checkpoints and that further studies on pH selectivity may provide new avenues for the development of immunotherapeutic drugs Fig. 1.

## The expression and function of PSGL-1 and VISTA

### PSGL-1

PSGL-1 is an inhibitory molecule that is widely expressed in hematopoietic cells, including myeloid and lymphoid cells, as well as platelets [45]. Historically, researchers have focused on the role of PSGL-1 in regulating cell migration [46]. Recently, studies have revealed that PSGL-1 is not only an adhesion molecule that recruits T cells into inflammatory tissues but has also been shown to be involved in other functions, including chemokine binding, signal transduction, tolerance induction, and T cell exhaustion.

In lymphocytes, PSGL-1 is initially expressed in a nonfunctional form on T cells until modified by glycosyltransferases during T cell activation [47–49]. PSGL-1 has multiple functions, including inhibiting effector T cell proliferation, promoting T cell depletion, limiting their survival and regeneration, decreasing memory T cell production, and promoting Regulatory cell (Treg) immunomodulation. In vitro studies have shown that PSGL-1 negatively regulates the proliferation of stimulated T cells by inhibiting T-cell receptor (TCR) signaling, reducing IL-2 signaling and cell survival, and upregulating other inhibitory receptors [50, 51]. PSGL-1 also inhibits the regenerative potential of CD4^+^ and CD8^+^ T cells depleted during ICI treatment [52]. Moreover, cross-linking of PSGL-1 by anti-PSGL-1 monoclonal antibody (mAb) results in death signaling in activated T cells [53]. In addition to its inhibitory effects on effector T cells, PSGL-1 deficiency leads to an increased frequency of memory T cells in mice infected with viruses [54]. Furthermore, Tregs express PSGL-1, and interaction with platelet P-selectin leads to the release of anti-inflammatory mediators IL-10 and TGF-β from Tregs. This interaction helps shift the immune environment towards an inhibitory state [55].

In myeloid cells, PSGL-1 is consistently expressed in a functional form on neutrophils, monocytes, and dendritic cells (DCs). PSGL-1 expression on DCs has been shown to induce the formation of tolerogenic DCs. These DCs increase the production of CD4^+^Foxp3^+^ Tregs within the thymus and diminish TCR signaling, thereby promoting immunosuppression [56]. When PSGL-1 is expressed on macrophages (Mø), antagonist anti-PSGL-1 antibody treatment leads to a decrease in anti-inflammatory phenotypic M2-type [57]. Furthermore, aggregates formed by platelets interacting with PSGL-1-expressing Tregs can promote Mø transcriptional reprogramming and polarization towards M2, thereby contributing to inflammatory remission [55]. These studies reveal that PSGL-1 contributes to an immunosuppressive microenvironment in myeloid cells by promoting tolerogenic DCs and M2. This regulation affects T cell proliferation, depletion, survival, function, and Tregs function.

### VISTA

VISTA is a negative checkpoint regulator molecule (NCR) significantly expressed in monocytes, Mø, DCs, and various types of T cells, but not in B lymphocytes or natural killer (NK) cells. VISTA is not only expressed as a ligand on myeloid cell to regulate the production of cytokines, chemotaxis, and phagocytosis, and to help them differentiate towards tolerogenic phenotype, but also as a receptor on T cell to participate in T cell quiescence, inhibit T cell activation. In the tumor microenvironment (TME), VISTA is highly expressed on myeloid-derived suppressor cells (MDSCs) and regulates their effector functions. Recent studies have also observed VISTA expression on tumor cells, such as those in melanoma and endometrial cancer [58].

VISTA can be expressed directly on T cells as a co-inhibitory receptor [34]. Studies have shown that CD4^+^ T cells from VISTA-deficient mice exhibit a markedly enhanced response to antigenic stimulation [34]. VISTA is also highly expressed on Foxp3^+^ Tregs. The use of a blocking VISTA antibody has been demonstrated to inhibit natural Treg differentiation and function while enhancing T cell proliferation, activation, and effector functions [35]. In addition, VISTA maintains the iTregs pool size by promoting iTregs differentiation and preventing their transformation into other CD4^+^ T cell subsets [59]. These studies were based on the use of monoclonal antibodies to VISTA. Another study demonstrated that direct knockdown of the VISTA gene resulted in the gradual accumulation of spontaneously activated T cells and the production of a range of inflammatory cytokines and chemokines [60]. These findings indicate that constitutive expression of VISTA correlates with the initial activation threshold of T cells. A recent study defined VISTA as the earliest peripheral T cell tolerance checkpoint regulator identified to date, which functions to enforce quiescence in naïve T cells, and this regulation of quiescence is inextricably linked to the ability to maintain self-tolerance in T cells that have not been negatively selected for [61].

Except for its inhibitory function by expression on T cells, VISTA can be highly expressed on APCs as a ligand, thereby inhibiting T cell proliferation and cytokine production upon interaction with receptors on T cells [35]. VISTA dampened TLR mediated activation of MAPK/AP-1 and IKK/NF-κB signaling cascades. At cellular levels, VISTA regulated the effector functions of MDSCs and tolerogenic DCs to promote their inhibitory function on T cells [62]. VISTA mediates MDSC function, and VISTA knockdown alleviates MDSC-mediated T-cell suppression [63]. Furthermore, VISTA plays a pivotal role in the differentiation of MDSCs. Deficiency of VISTA has been observed to result in a reduction in STAT3 activation and STAT3-dependent polyamine production, which impairs MDSC amplification [64]. Additionally, targeting VISTA on Mø with agonistic antibodies leads to their reprogramming towards a tolerogenic M2-type phenotype, inducing anti-inflammatory programs [65]. Moreover, increased expression of genes related to the cell cycle and immune activation has been observed in microglia from VISTA knockout (KO) mice [66]. It has also been found that beyond membrane-type VISTA, human peripheral monocytes continuously produce and release soluble VISTA, which impairs the cytotoxic activity of T cells but does not induce their programmed death [67].

**Fig. 2:**
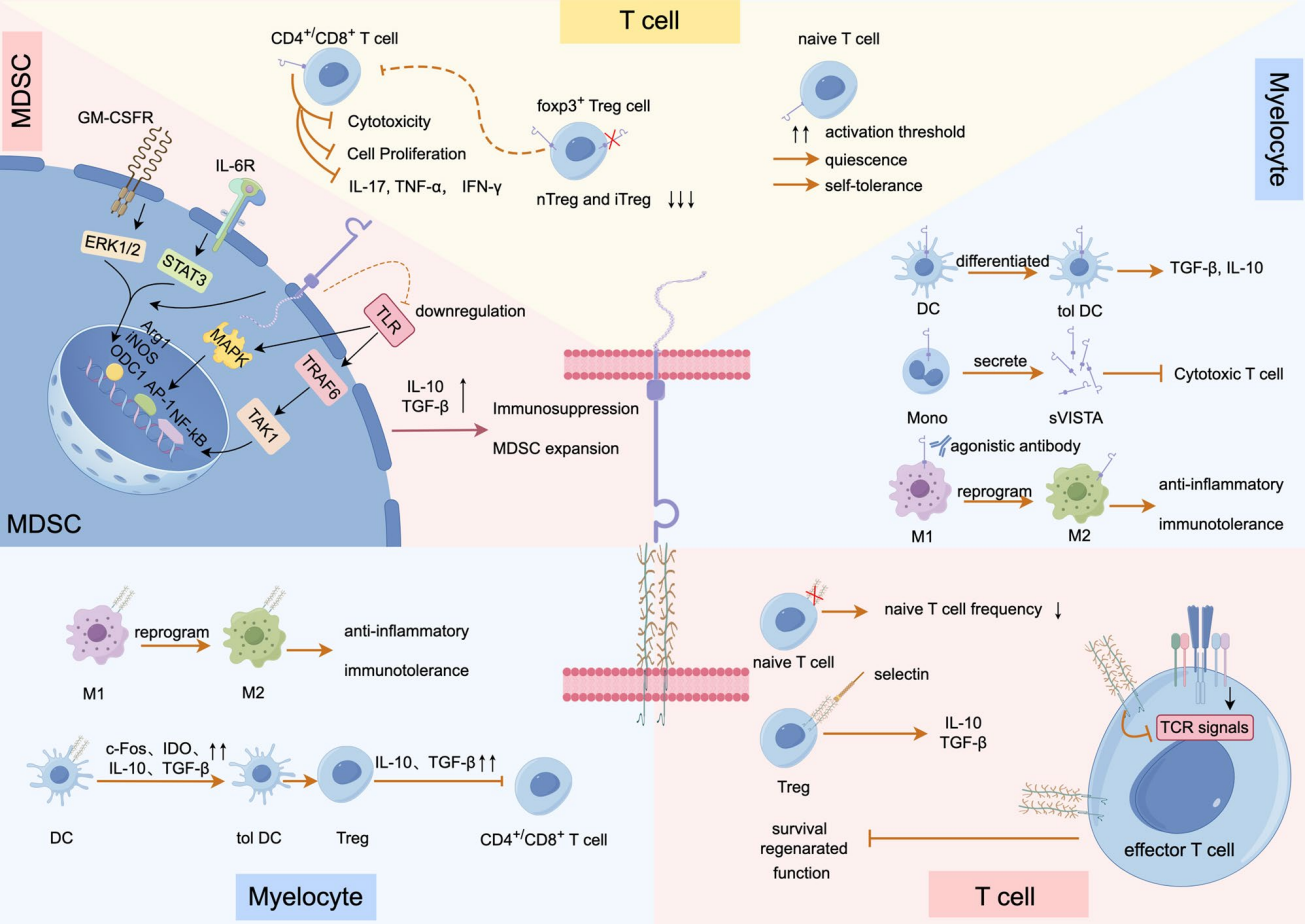
The Expression and Function of PSGL-1 and VISTA. PSGL-1 and VISTA are a pair of immune checkpoints that are widely expressed in hematopoietic cells, including myeloid and lymphoid cells. PSGL-1 has multiple functions in lymphocytes, including inhibiting the proliferation of effector T cells, promoting T cell depletion, limiting their survival and regeneration, decreasing the frequency of memory T cell production, and promoting Tregs immunomodulation. In myelocytes, PSGL-1 expression on DCs and macrophages has been shown to induce the formation of tolerogenic DCs and M2. VISTA is not only expressed as a ligand on myeloid cells to regulate the production of cytokines, chemotaxis, and phagocytosis, and to help them differentiate towards a tolerogenic phenotype, but also as a receptor on T cells to participate in T cell quiescence, inhibit T cell activation. Furthermore, VISTA is highly expressed in MDSCs to promote immunosuppression. (By Figdraw)

Both PSGL-1 and VISTA are highly expressed in hematopoietic cells and exert regulatory effects on their function. In myeloid cells, both PSGL-1 and VISTA have the capacity to facilitate their transformation into a tolerogenic phenotype, thereby inducing an immune-tolerant microenvironment. In T lymphocytes, PSGL-1 and VISTA can directly or indirectly inhibit T cell proliferation and activation, thereby attenuating cellular immune effects and establishing an immunosuppressive microenvironment. Therefore, further mechanistic studies of PSGL-1 and VISTA in myeloid cells and T cells could uncover novel therapeutic targets for enhancing immune function (e.g., in cancer) or suppressing immune responses (e.g., in autoimmune diseases and transplant rejection) Fig. 2.

## The potential of VISTA and PSGL-1 in multiple diseases

### PSGL-1 and VISTA play a role in the negative regulation of autoimmunity

**Fig. 3:**
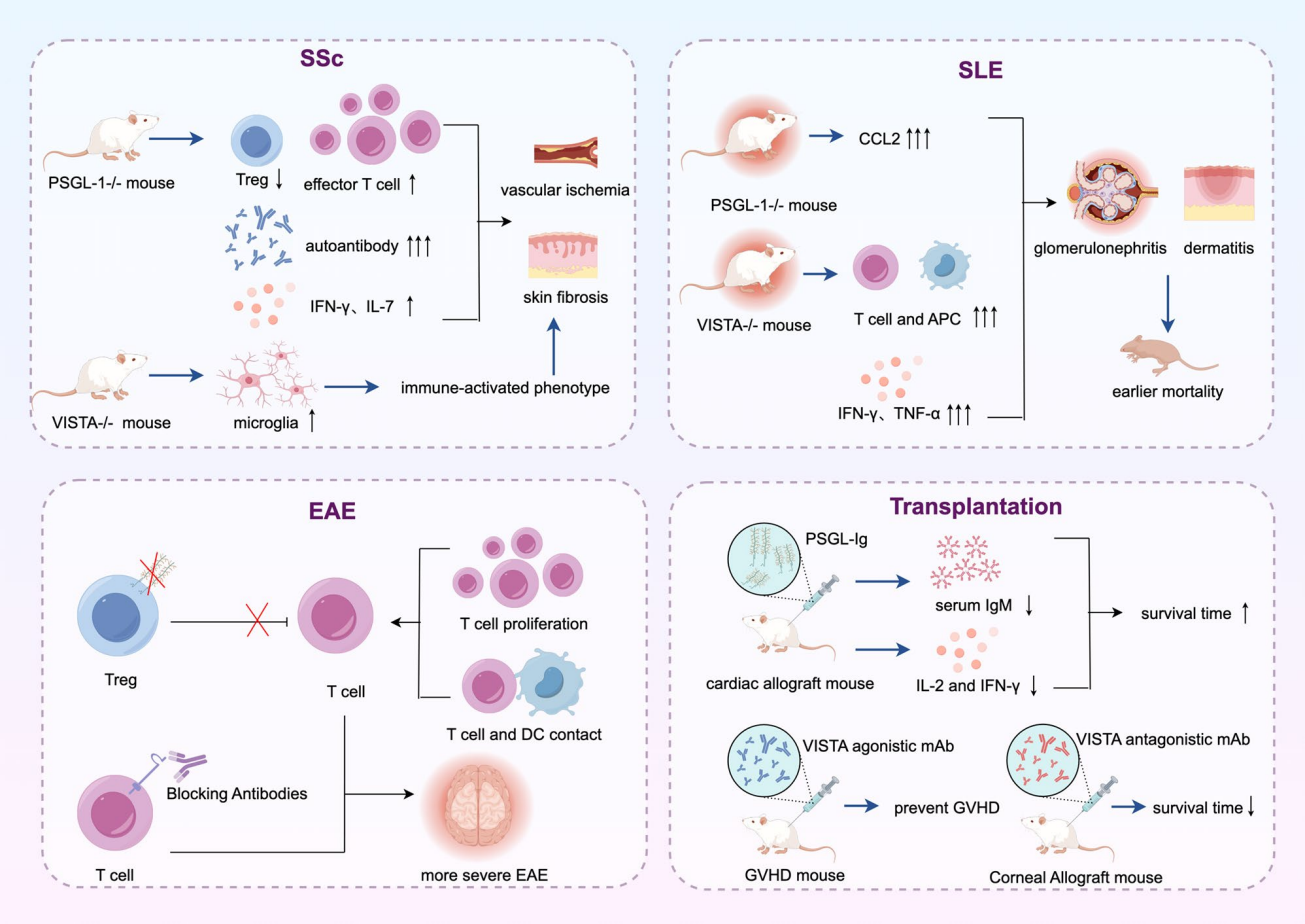
PSGL-1 and VISTA in the regulation of autoimmune diseases and transplantation tolerance. PSGL-1 and VISTA play a vital role in the negative regulation of autoimmune disease and transplantation tolerance induction. In autoimmune diseases and transplant rejection, PSGL-1 can maintain immune tolerance by modulating Tregs function and preventing excessive activation of T cells. VISTA helps maintain T cell quiescence and regulate immune tolerance. PSGL-1 and VISTA deficiency results in enhanced T cell activation and exacerbates autoimmune reactions and transplant rejection, such as SSc, SLE, EAE, GVHD, and Corneal transplant rejection. (By Figdraw)

Autoimmune diseases arise when the immune system mistakenly targets self-tissues, leading to excessive immune responses. Immune checkpoint molecules play a crucial role in maintaining self-tolerance and preventing excessive immune responses. In autoimmune diseases, PSGL-1 can maintain immune tolerance by modulating Treg function and preventing excessive activation of T cells. VISTA helps maintain T cell quiescence and regulate immune tolerance. PSGL-1 and VISTA deficiency results in enhanced T cell activation and exacerbates autoimmune conditions, such as systemic sclerosis (SSc), systemic lupus erythematosus (SLE), and experimental autoimmune encephalitis (EAE) Fig. 3.

### Systemic sclerosis (SSc)

It has been found that skin-resident immune cells in PSGL-1 -/- mice have a pro-inflammatory phenotype, with a disproportionate ratio of T effector to Tregs, and that mouse serum contains circulating autoantibodies commonly found in human autoimmune disorders, which ultimately leads to overactivation of the immune system triggering an autoimmune disease similar to SSc [68]. Furthermore, studies have been conducted on the complications of SSc. For instance, pulmonary arterial hypertension (PAH), one of the complications of SSc, where PSGL-1-deficient mice have increased numbers of T cells and B cells, increased production of IFN-γ, and decreased numbers of Tregs which in turn accelerated the development of PAH, making the condition of spontaneous SSc-like syndrome worse [69]. Additionally, PSGL-1 has been demonstrated to influence the development of interstitial pneumonia (ILD), a further complication of SSc. PSGL-1-/- mice have exhibited augmented inflammatory cell infiltration and increased expression of pro-inflammatory and pro-fibrotic cytokines, resulting in elevated interstitial and peribronchial fibrosis [70]. The role of VISTA in SSc has also been documented. A study by Borggrewe revealed that VISTA expression was diminished in microglia in multiple sclerosis lesion tissues, and this study pioneered the observation that microglia express VISTA in the central nervous system [66].

### Systemic lupus erythematosus (SLE)

PSGL-1-deficient lupus mice increased renal CCL2 expression, which in turn accelerated the progression of lupus nephritis and resulted in earlier mortality [71]. This indicates that PSGL-1 has the potential to limit the production of inflammatory chemokines and reduce the chemotactic effect on inflammatory cells. More studies have been conducted to investigate the role of VISTA in the pathogenesis of SLE. A study by Ceeraz demonstrated that VISTA-/- mice resulted in enhanced activation of splenic CD4^+^ T cells and myeloid cell populations, and pro-inflammatory cytokines, chemokines, and IFN-γ regulated genes associated with SLE were elevated in these mice, which contributed to the aggravation of SLE [36]. Another study revealed that VISTA-/- mice spontaneously develop cutaneous and systemic autoimmune disorders similar to human lupus [72]. Yang also found a similar phenomenon, where VISTA deficiency exacerbated lupus-like disease in mice, which was associated with aberrant activation of type I interferon (IFNα) signaling, CD4^+^ T-cells, and the NF-κB pathway [73].

### Autoimmune encephalitis (EAE)

He et al. found that Tregs from mice lacking PSGL-1 were unable to limit T cell proliferation and their interaction with DCs during the late stages of T cell activation, and were also unable to inhibit T cell proliferation in lymph nodes, making PSGL-1-/- mice suffer more severely than WT mice [74]. In addition to the inhibitory effect of PSGL-1 on EAE, VISTA expression decreased in mouse microglia [75]. Wang discovered that VISTA expression on APCs inhibits T cell proliferation and cytokine production in vitro and that a VISTA-specific monoclonal blocking antibody disrupted the suppression of T cell response by VISTA-expressing APCs, resulting in exacerbated T cell-mediated EAE in mice [42].

#### PSGL-1 and VISTA can induce transplantation immune tolerance

The majority of existing allograft therapies ultimately result in immunodeficiency and an increased risk of recurrence of malignancies and infections [76]. With this in mind, the future direction of transplantation therapy is to induce graft tolerance within the organism. Thus, targeting activation of inhibitory immune checkpoints such as PSGL-1 and VISTA may represent a novel strategy for conferring immune privileged status to other organs, to suppress allograft rejection.

Coito to observed that infusion of a soluble recombinant form of PSGL-1 (rPSGL-Ig) during skin graft-mediated sensitization prevented “accelerated” rejection and prolonged cardiac allograft survival [77]. In addition, Deng discovered the pathogenesis of GVHD is related to the expansion of extrafollicular PSGL-1^low^CD4^+^ T cells, and blockade of donor CD4^+^ T cells by ICOS, BCL6, or STAT3 prevents the expansion of PSGL-1^low^CD4^+^ T cells, which would improve the induction of GVHD [78]. Moreover, Flies also demonstrated that VISTA antibodies could effectively modulate allogeneic T cell responses while simultaneously inhibiting T cell aggregation and expansion in GVHD target organs [41]. Subsequently, his team also identified two distinct mechanisms by which VISTA induces T cell tolerance in transplantation. First, signaling through the VISTA co-suppressor receptor effectively prevents the activation and expansion of allo-responsive donor T-cells during the initiation phase. Second, donor Tregs subsequently expand to maintain long-term tolerance and GVHD suppression [24]. Another study found that constitutively expressed VISTA plays a role in allogeneic-specific anterior chamber-associated immune deviation (ACAID), inhibiting T cell infiltration in corneal transplantation [79].

However, the exact mechanism by which PSGL-1 and VISTA regulate T cell responses remains to be elucidated. Consequently, further studies are required to identify PSGL-1 and VISTA-mediated APC and T-cell signaling pathways, to manipulate the immune system in a way that maintains tolerance and graft survival while minimizing side effects Fig. 3.

### PSGL-1 and VISTA help tumor immune escape

**Fig. 4:**
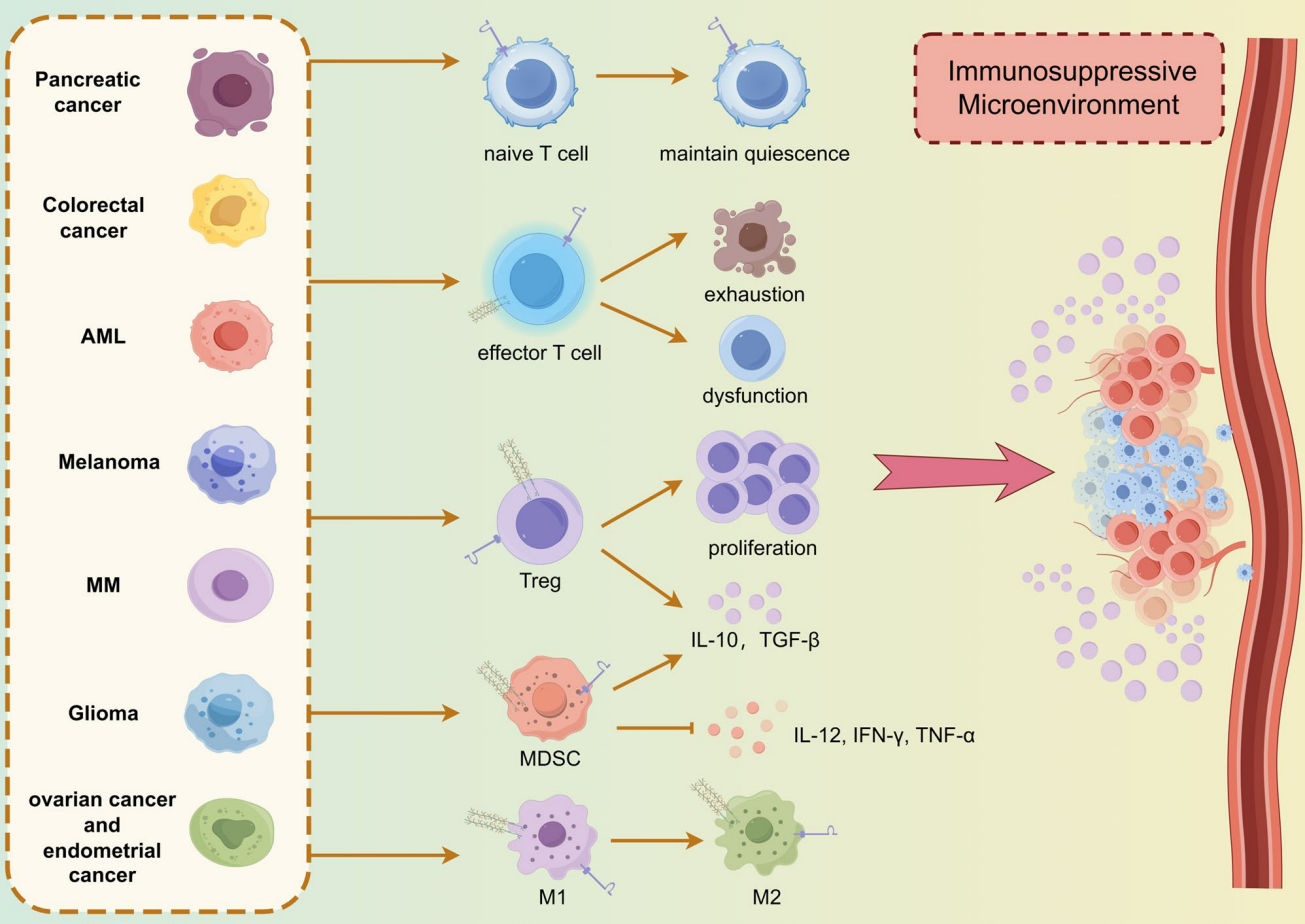
PSGL-1 and VISTA in tumor immune escape. The pH of the TME is 5.85-6 and PSGL-1 and VISTA can stably bind under acidic conditions. They influence many different cells in the TME to create an immunosuppressive milieu that facilitates tumor immune evasion and exacerbates disease progression in a multitude of cancers, including MM, AML, Glioma, Melanoma, Colorectal cancer, Pancreatic cancer, ovarian cancer and endometrial cancer. This is exemplified by their capacity to promote the differentiation of tumor-associated macrophages into M2, the proliferation and Inhibitory function of MDSCs and Tregs, the maintenance of naive T cells quiescence, and the depletion of effector T-cells. (By Figdraw)

Several studies have demonstrated that VISTA shows high expression in TME and creates an Immunosuppressive microenvironment that impairs the efficacy of protective anti-tumor immunity [42]. One study has indicated that PSGL-1 and VISTA can stably bind under acidic conditions [18]. It is known that there are several types of immune cells in TME, including tumor-associated macrophages (TAMs), Tregs, and MDSCs, which are associated with acidic, hypoxic, and inflammatory cell-rich environments [80]. And the pH of the TME is 5.85–6.5 [81]. It is therefore reasonable to hypothesize that under the low pH conditions commonly found in tumors, the PSGL-1 and VISTA pathway suppresses immune activation and promotes tumor immune escape Fig. 4.

### Melanoma

Tinoco discovered that PSGL-1 deficiency in a melanoma model resulted in PD-1 downregulation, which led to enhanced T cell responses and Inhibited tumor progression [50]. DeRogatis similarly observed in a melanoma model that PSGL-1-deficient mice exhibited augmented infiltration of CD4⁺ and CD8⁺ T cells, accompanied by elevated production of anti-tumor factors such as IFN-γ and TNF-α, resulting in delayed tumor growth [50]. Moreover, TAMs underwent a pro-inflammatory shift following anti-PSGL-1 antagonist antibody treatment in a humanized melanoma model, and a comparable phenomenon was observed in ex vivo tumor cultures from patients [57]. These studies demonstrate that PSGL-1 plays an important role in melanoma development. VISTA also represents a significant regulatory molecule in melanoma. In a B16 melanoma model, blockade of VISTA altered the suppressive cellular profile of TME and reduced tumor-infiltrating MDSCs, while increasing the frequency of infiltrating effector T cells and enhancing the immunostimulatory phenotype of DCs with higher levels of expression of MHC II and CD80, and greater production of cytokines IL-12 and TNF-α [35]. Except for its inhibitory effect on immune cells within the melanoma microenvironment, VISTA can be directly expressed on melanoma cells, thereby promoting tumor escape and progression. Li observed that HIF-2α mediated an increase in the expression of VISTA on melanoma cells, which may interact with the PSGL-1 receptor on T cells, thereby inhibiting T cell function and promoting tumor immune escape [58]. Rosenbaum also found that VISTA showed high expression in melanoma patient samples and cell lines which is negatively regulated by FOXD3, and increased intra-tumoral Tregs and enhanced PD-L1 expression on TAMs [82]. Additionally, VISTA has the potential to serve as a prognostic marker for advanced melanoma. Kuklinski analyzed 85 primary melanoma specimens from the clinic and found that the presence of VISTA was associated with significantly poorer disease-specific survival, suggesting that VISTA is an independent negative prognostic factor in primary cutaneous melanoma [83].

### Pancreatic adenocarcinoma (PAAD)

The immunosuppressive microenvironment of pancreatic tumors is primarily attributed to MDSCs. Lu first reported that PSGL-1 was expressed on the surface of MDSCs in pancreatic tumor tissues and that the P-selectin/PSGL-1 binding could promote the recruitment of MDSCs to pancreatic tumor tissues, which in turn creates a tumor-suppressive microenvironment [84]. In conjunction with the prominent effect of PSGL-1 in PAAD, VISTA exerts a comparable inhibitory effect. Liu found that VISTA was expressed mainly in high-density infiltrating immune cells but rarely in human pancreatic cancer cells by immunohistochemical analysis of human pancreatic cancer samples [85]. Additionally, studies at the RNA level have yielded comparable results. Nishizaki utilized RNA sequencing analysis and observed that high VISTA RNA expression was present in 32% of tumors, representing the most prevalent checkpoint for high expression across nine evaluated tumors, and high VISTA expression was associated with poor survival following immunotherapy for PAAD [86]. Subsequently, Rezagholizadeh also found elevated levels of VISTA in pancreatic cancer samples compared to normal tissues, indicating that VISTA could have potential as a diagnostic and prognostic indicator for PAAD [87].

### Colorectal cancer(CRC)

In a colorectal cancer model, PSGL-1 on TAM could bind to P-selectin and activate the JAK/STAT1 pathway, inducing the transcription of C5 and the release of C5a, which shifted the TAM to the M2 phenotype-mediated tumor-suppressor microenvironment, and that the blockade of PSGL − 1 significantly reduced the growth of colorectal cancer [88]. Notably, VISTA also plays a significant role in colorectal cancer. In the CT26 colorectal cancer model, tumors presented complete adaptive resistance to anti-PD-1/CTLA-4, but the addition of anti-VISTA increases contact between T cells and myeloid cells, and reduces regulators of T cell quiescence, leading to clearance of half of the tumors [89]. Deng demonstrated that hypoxia-inducible factor (HIF)-1α binding to the conserved hypoxia-responsive element in the VISTA promoter could upregulate VISTA expression on MDSCs, and that antibody targeting of VISTA under hypoxic conditions could alleviate MDSC-mediated T-cell suppression and promote anti-tumor responses [63].

### Glioma

Yuan performed snRNA-Seq and spatial transcriptomics (ST) analyses on untreated glioma samples and found that both VISTA and PSGL-1 were highly expressed in golima, and the high expression of VISTA in glioma was associated with poorer patient prognosis [90]. Ghouzlani additionally discovered that VISTA may be linked to glioma progression. VISTA was more strongly expressed in high-grade glioms with weaker overall survival and its expression was positively correlated with that of the traditional immune checkpoint PD-1^[99]^. These studies indicate that PSGL-1 and VISTA have the potential to serve as diagnostic markers for glioma, in addition to offering a basis for considering targeted therapies, which may also provide new avenues for glioma patients.

### Ovarian cancer (OV) and endometrial carcinoma (EC)

Plasma PSGL-1 expression is markedly elevated in patients with epithelial ovarian cancer, rendering it a reliable biomarker for differentiating between healthy individuals and those with epithelial ovarian cancer [91]. With regard to VISTA in human endometrial cancer, it was demonstrated that the co-expression of VISTA and ARG1 on tumor-associated myeloid cells was associated with poor patient survival [64]. Mulati even demonstrated for the first time that VISTA was highly expressed in human ovarian and endometrial cancers, and that the expression on tumor cells of VISTA can inhibit T cell proliferation and cytokine production in vitro and reduce CD8^+^ T cell infiltration in vivo, and anti-VISTA antibody treatment prolonged the survival time of tumor-bearing mice [92].

### Acute myeloid leukemia (AML)

RUNX1/ETO(RE) oncoprotein is a leukemia transcription factor associated with the development of t(8;21) ^+^ AML, which binds to the PSGL-1 promoter region to negatively regulate PSGL-1 expression [93]. The differential expression of PSGL-1 during myeloid hematopoietic development and the quantification of PSGL-1 can be employed as a marker of the immunophenotype of AML subpopulations, with greater expression observed in myeloid cells and less in monoblasts [94]. These studies demonstrated that PSGL-1 can be utilized as a marker for the clustering of AML subpopulations. Studies on VISTA and AML have revealed that STAT3 directly binds to the DNA-responsive element of the VISTA gene to promote high VISTA expression on AML cells and this high expression has also been associated with a poor prognosis in patients with AML [95]. In a mouse model of humanized AML, overexpression of VISTA has been observed to facilitate tumor cell evasion and promote AML cell growth [96]. Conversely, antibody blockade or knockdown of VISTA has been demonstrated to enhance T cell activity, thereby significantly inhibiting AML progression [96]. Beyond its direct expression on AML cells to promote tumor immune escape, VISTA is also highly expressed on MDSC in the peripheral blood of AML patients, and specific knockdown of VISTA effectively reduces the inhibition of CD8^+^ T cell activity by MDSCs and promotes anti-tumor responses [97].

### Multiple myeloma (MM)

Azab demonstrated that PSGL-1 expression on MM cells not only regulates integrin activation and downstream signaling but also enables MM cells to develop drug resistance [98]. Huang also identified a close relationship between VISTA and MM progression. They observed that VISTA, along with other immune checkpoints PD-1, Tim-3, and TIGIT, is highly expressed in MM patients’ alveolar T cells. This leads to T cell exhaustion and dysfunction, which are associated with disease progression and clinical indicators [99]. In light of these findings, VISTA may be regarded as a prospective target for the reversal of T cell exhaustion and the enhancement of T cell functionality.

## Targeting VISTA/PSGL − 1 for multiple treatment modalities

Immune checkpoint blockade therapies have yielded remarkable success in a number of cancer patients. However, some patients do not respond to current immunotherapies, indicating a need for the identification, development, and testing of additional targets. Moreover, apart from their application to cancer, therapies targeting immune checkpoints have the potential to be extended to multiple disease areas, including autoimmunity and transplantation. In contrast to CTLA-4, which inhibits activation during the initial phase, and PD-1, which exerts an inhibitory function during the effector phase, VISTA is consistently expressed on naive T cells that maintain peripheral T cell quiescence at the earliest possible stage, making it a highly promising therapeutic target. Nevertheless, PSGL-1 has the potential to impact a multitude of inhibitory pathways, rendering it an appealing candidate for the more expansive targeting of inhibitory mechanisms to alleviate T cell dysfunction. Currently, a variety of therapeutic approaches have been developed, including monoclonal antibodies (mAbs), small molecule peptides and their analogs, combinations of multiple immune checkpoints, and others Table 1.

**Table 1:**
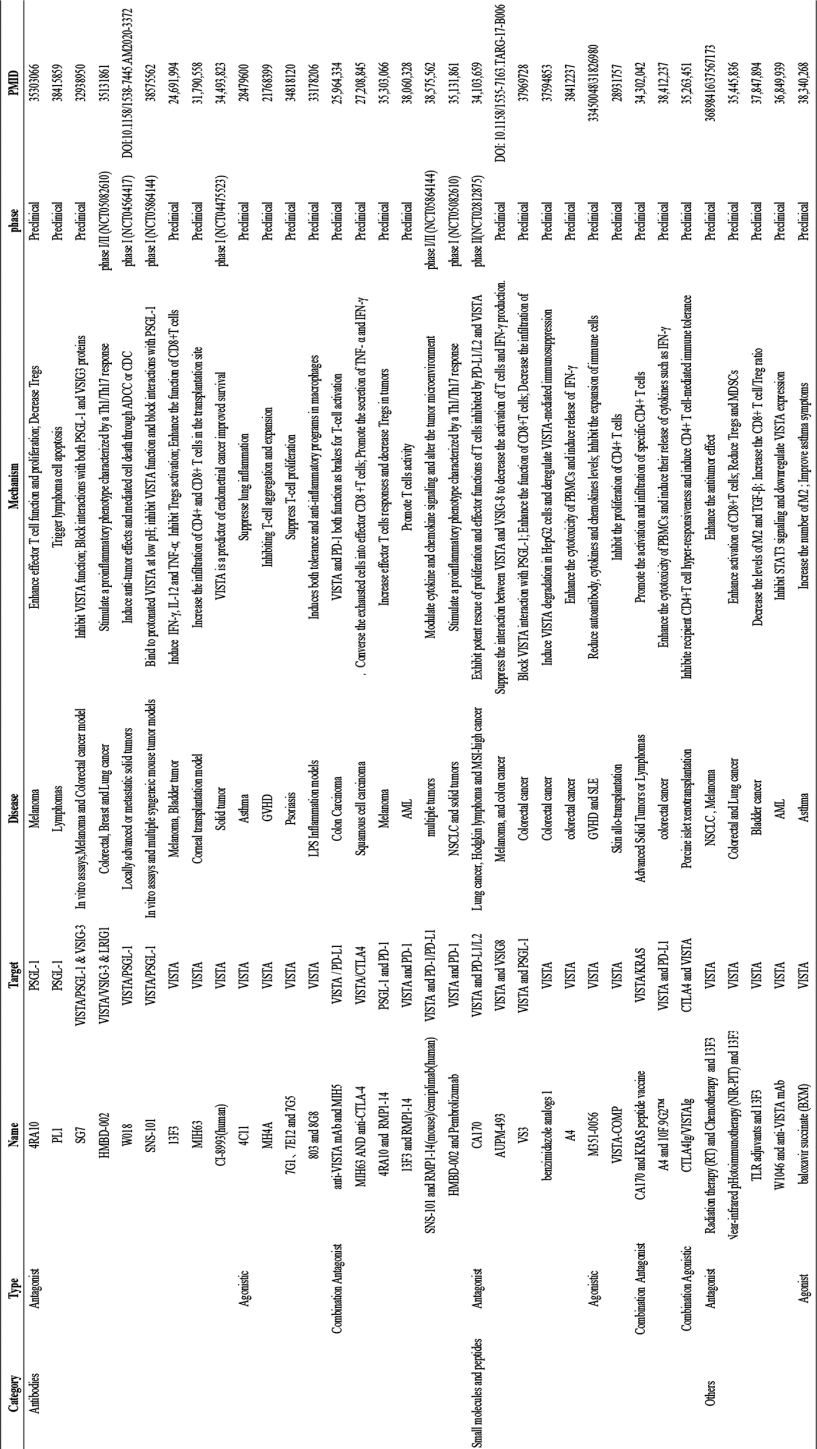
Current targeting PSGL-1 and VISTA therapeutics and clinical developement

### Antibody

Since the introduction of rituximab in 1997 [100], Monoclonal antibody drugs have experienced rapid growth and shown remarkable performance in tumor therapy due to their high specificity, strong affinity and long serum half-life [101]. As the pair of PSGL-1 and VISTA, a new checkpoint molecule, has gradually come to the attention of researchers, related targeted monoclonal antibodies have also been the subject of intensive investigation.

### Antagonistic antibodies

DeRogatis showed that a therapeutic anti-PSGL-1 antibody(4RA10) increased the proliferation and function of effector T cells while reducing the number of Tregs, which slowed tumor growth [22]. Another anti-PSGL-1 antibody(PL1) could decrease cell viability and trigger apoptosis in lymphoma cells [102]. More relevant antibodies targeting VISTA were studied. Anti-VISTA mAb(13F3) treatment increased the number, infiltration, and effector function of tumor-specific T cells, and altered the suppressive feature of the TME by decreasing the presence of MDSC and tumor-specific CD4^+^Foxp3^+^ Tregs [35]. In a mouse corneal transplantation model, the action of the antagonistic anti-VISTA antibody(MIH63) has been observed to increase the infiltration of the transplantation site by CD4^+^ and CD8^+^ T cells, which in turn has been shown to lead to a decrease in graft survival [79]. Then, Mehta devised an anti-VISTA antibody (SG7) that exhibits a high affinity for mouse, human, and monkey VISTA, effectively blocking its interaction with PSGL-1, inhibiting the function of VISTA, and enhancing the survival rate of melanoma-bearing mice [103]. Subsequently, a study by Mostböck demonstrated that anti-VISTA antagonistic antibodies exert their effects dependent on interaction with different Fc receptors, and anti-VISTA antibodies without the FC segment have little or no effect in any model system [104]. In light of this report, Thakkar developed the first Fc-independent anti-VISTA blocking antibody( HMBD-002), which binds to a functional epitope within the predicted C-C loop and functions through stimulation characterized by the Th1/Th17 response, and this mechanism of function is further supported by the use of this antibody in humanized mouse models of colorectal, breast, and lung cancer [105]. In general, these VISTA antibodies are subject to clearance issues and the potential for cytokine release syndrome, which impede their effective targeting. In a recent development, anti-VISTA mAb with IgG1 subtype(W018) developed by Pierre Fabre, inhibits PSGL-1 binding to VISTA at pH 6–7.4 [106]. Thisted also has created a novel antibody(SNS-10), which is pH-selective in its binding to VISTA. In preclinical trials, pH-selective VISTA antibodies have demonstrated a superior safety profile in non-human primates when compared to non-pH-selective antibodies [107]. The development of pH-selective VISTA and other antibodies represents a promising new avenue for cancer therapy.

#### Agonistic antibodies

Tinoco initially demonstrated that the VISTA agonistic antibody (MH4A) potently modulated allogeneic T-cell responses while inhibiting T-cell aggregation and expansion in GVHD target organs [41]. the VISTA agonist antibody (4C11) suppressed lung inflammation and reduced the severity of the disease [108]. ElTanbouly demonstrated that VISTA agonistic antibodies, anti-human VISTA clone 803 and anti-mouse VISTA clone 8G8, could maintain the anti-inflammatory properties of Mø, thereby enhancing tolerance to LPS in mice [65]. Ma developed a series of agonistic anti-VISTA mAbs,7G1, 7G5, and 7E12, which target VISTA in mice, humans, or both, and agonize VISTA and attenuate inflammation in a murine psoriasis model [109].

### Small molecules and peptides

The field of monoclonal antibody technology is constantly evolving, yet there are still numerous limitations to be overcome. These include the relatively poor conformational stability, Limited ability to penetrate and accumulate Organizations and the potential for immunogenicity [110]. In light of these challenges, synthetic peptides that are designed to bind to specific antigens represent a promising alternative to antibodies.

#### Antagonistic small molecules and peptides

CA-170, an amino acid-inspired small molecule inhibitor of PD-L1 and VISTA, exhibited potent rescue of proliferation and effector functions of T cells inhibited by PD-L1 and VISTA [111]. Interestingly, Musielak and his colleagues demonstrated that CA-170 did not bind to mouse PD-L1 or human PD-1 either, and it may act downstream from the PD-1 receptor or on any other T cell-activating pathway [112]. So, we should also remain skeptical about CA-170 targeting VISTA. A small molecule (AUPM-493) developed by Aurigene and acts as a PD-L1 and VISTA antagonist, led to the activation of T cells and IFN-γ production [113]. Niu developed a pH-selective peptide drug (VS3), which binds to VISTA with high affinity and blocks its interaction with the ligand PSGL-1 under acidic conditions, thereby enhancing the function of CD8^+^ T-cells and decreasing the infiltration of MDSCs [114]. Meanwhile, benzimidazole analogs 1 could act as a VISTA bifunctional inhibitor, which could both bind to VISTA to induce VISTA degradation in HepG2 cells and also deregulate VISTA-mediated immunosuppression, and significantly inhibit tumor growth in a mouse CT26 model [115]. Small molecule inhibitor A4, with a high affinity for VISTA proteins, enhanced the cytotoxicity of PBMCs against tumor cells by activating PBMCs and inducing their release of cytokines such as IFN-γ [116].

#### Agonistic small molecules and peptides

Yang showed that the VISTA small molecule agonist M351-0056 can reduce autoantibody inflammatory cytokines and chemokines levels, and even inhibit the expansion of immune cells in cGVHD mice and MRL/lpr mice [73]. Additionally, Hu proved that the compound M351-0056 exhibited a high affinity for VISTA, which could enhance the VISTA-mediated immunosuppressive pathway [117]. Prodeus devised a stable pentameric VISTA construct (VISTA-COMP) by genetically fusing the IgV structural domain of VISTA with the pentameric structural domain of cartilage oligomeric matrix protein (COMP), which can inhibit the proliferation of polyclonally activated CD4^+^ T cells without immobilization as an immunosuppressive agonist and prolong the survival time of skin allografts in a mouse transplantation model [118].

#### Combined action with other immune checkpoint antibodies and small molecules and peptides

During clinical treatment of cancer, it has been found that singularly targeting a particular checkpoint leads to a compensatory elevation of the expression of other inhibitory checkpoints, thus rendering the targeted therapy ineffective [119]. Therefore, a combination immunotherapy approach targeting multiple immune checkpoints is warranted.

Liu demonstrated that VISTA plays a non-redundant role in controlling T-cell activation by using a combined blockade with a mAb specific for VISTA and PD-L1, which resulted in optimal colorectal cancer treatment [120]. Schaafsma uncovered that large CT26 tumors showed complete adaptive resistance to anti-PD-1/CTLA-4, but the addition of anti-VISTA upregulated the stimulated antigen-presentation pathway and reduced myeloid-mediated inhibition, resulting in the rejection of half of the tumor rounds [89]. Kim observed that VISTA blockade in combination with anti-PD-1 therapy produced synergistic anti-leukemic effects [96]. With the development of pH-selective antibodies, Thisted developed a new pH-selective VISTA antibody, SNS-101, which also showed improved efficacy in combination with PD-1 inhibitors, modulating cytokine and chemokine signaling and altering TME [107]. In addition, the CTLA-4 and VISTA antibody combination efficiently inhibited Treg recruitment and increased the ratios of both CD8^+^ T/ Treg and CD4^+^ conventional T (Tcon)/Treg in the Squamous cell carcinoma [121]. About PSGL-1, DeRogatis proved that co-targeting PSGL-1 and PD-1 also enhanced anti-tumor immunity and slowed down melanoma tumor growth [22].

For small molecule peptide drugs, the KRAS peptide vaccine designed by Pan has been demonstrated to promote the activation and infiltration of specific CD4^+^ T cells. When used in combination with CA-170, the vaccine has been shown to enhance the efficacy of the treatment regimen [122]. Sun synthesized A4, which also synergistically enhances the anticancer efficacy with PD-L1 antibody [116]. Except for its application in tumor therapy, Wang found that the combination of CTLA4-Ig and VISTA-Ig could selectively inhibit the hyperreactivity and pro-inflammatory cytokine production of CD4^+^ T cells in porcine pancreatic islet transplantation recipients by SOCS1-dependent signaling, which significantly delayed xenograft rejection [123].

### Others

Radiation therapy (RT) and Chemotherapy have long been the primary systemic treatment for many malignancies, making the combination of radiotherapy with immune checkpoint therapies a new therapeutic modality. Duval found that antagonist anti-VISTA antibodies significantly enhanced the antitumor effect of a single dose of 15 Gy radiation^[132^. Zhang found that VISTA blockade in combination with RT synergistically reduced immunosuppressive myeloid cells in their study of NSCLC [124]. Li demonstrated that chemotherapy promotes the overexpression of VISTA in melanoma cells, which facilitates tumor immune escape. The application of the VISTA-blocking antibody 13F3 enhanced the therapeutic effect of carboplatin, and this change was mediated by HIF-2α [58].

Near-infrared photoimmunotherapy (NIR-PIT) is a cancer treatment that utilizes an antibody-photo-absorbent (IRDye 700DX NHS ester) conjugate to selectively kill target cells after local application of NIR light. Upon combining anti-VISTA and NIR-PIT, enhanced activation of CD8 ^+^ T cells and DCs and reduced Treg and MDSC were observed in regional lymph nodes, which inhibited tumor cell progression and prolonged survival time in colorectal and lung cancer models in mice [125].

Antibodies can also be used in combination with TLR adjuvants. Wang found that in a synthetic mouse model of bladder cancer MB49, the combination of 13F3 and TLR3-specific adjuvant decreased the frequency and levels of M2 and the immunosuppressive molecule TGF-β1, upregulated the expression of immunostimulatory molecules and increased the CD8^+^ T cell/Treg ratio [126].

In combination with other molecular inhibitors, W1046 could significantly enhance the efficacy of VISTA mAb by inhibiting STAT3 signaling and downregulating VISTA. In addition, the combination of W1046 and VISTA mAb showed significant anti-AML effects in vitro and in vivo [95].

As well, new uses for old medicines are being developed. Di identified baloxavir succinate (BXM) as a VISTA agonist for the treatment of allergic asthma by performing a virtual screening of FDA-approved drugs, which binds directly to VISTA with high affinity, increases the number of M2 Mø, and can significantly improve asthma symptoms [127].

## Perspective

VISTA is expressed in T cells and myeloid cells, where it plays a key role in maintaining naïve T lymphocytes in a quiescent state [89]. PSGL-1, as a binding partner of VISTA, is also expressed on T cells and myeloid cells and can co-inhibit T cell activation when interacting with VISTA, especially under acidic conditions [50]. As a result, VISTA and PSGL-1 are promising candidates for immune checkpoint modulation. In addition to being a promising target for the treatment of cancer, PSGL-1 and VISTA are also a potential target for the treatment of autoimmune diseases and transplantation. Therefore, a variety of therapeutic approaches have been developed. Developing PSGL-1 and VISTA-specific antibodies or agonistic agents could suppress immune responses in autoimmune diseases and transplantation. Conversely, antagonistic or neutralizing agents could prevent PSGL-1 and VISTA from inhibiting signaling, thereby reversing T cell exhaustion and expanding treatment strategies in oncology. Additionally, the development of pH-selective antibodies targeting PSGL-1 and VISTA, along with combinations of other therapeutic modalities, may offer novel treatment options for patients who have not responded to current therapies.

Despite advancements in immune checkpoint therapy, the mechanisms and therapeutic potentials of newer checkpoints PSGL-1 and VISTA are not yet fully understood. Specifically, the in vivo interactions between PSGL-1 and VISTA and their roles in immune regulation remain to be elucidated. It is thus imperative that future research focus on exploring the signaling mechanisms of PSGL-1 and VISTA interactions, in particular how they regulate the function of T cells and myeloid cells, and their interactions with other immune checkpoints.

## Data Availability

No datasets were generated or analysed during the current study.

## Abbreviations

UC: Urothelial carcinoma
RCC: Renal cell carcinoma
NSCLC: Non-small-cell lung cancer
CRC: Colorectal carcinoma
HL: Hodgkin’s lymphoma
IrAEs: Immune-related adverse events
PSGL-1: P-selectin glycoprotein ligand-1
VISTA: V-domain immunoglobulin T-cell activation suppressor
GVHD: Graft-versus-host disease
PKC: Phosphokinase C
CK2: Casein kinase 2
Tregs: Regulatory cells
TCR: T-cell receptor
mAb: Monoclonal antibody
DCs: Dendritic cells
Mø: Macrophages
NCR: Negative checkpoint regulator
TME: Tumor microenvironment
MDSCs: Myeloid-derived suppressor cells
KO: Knockout
SSc: Systemic sclerosis
SLE: Systemic lupus erythematosus
EAE: Experimental autoimmune encephalitis
TAMs: Tumor-associated macrophages
CRC: Colorectal cancer
OV: Ovarian carcinoma
EC: Endometrial carcinoma
AML: Acute myeloid leukemia
MM: Multiple myeloma
PADD: Pancreatic adenocarcinoma
