# Correction: A pair of promising immune checkpoints PSGL-1 and VISTA from immunotolerance to immunotherapy

**DOI:** 10.1186/s40364-024-00709-3

**Published:** 2025-01-23

**Authors:** Manqing Peng, Xiaofang Lu, Junshuang Guo, Xiangli Yin, Jing Zhang, Xin Li, Yizhou Zou

**Affiliations:** Department of Immunology, School of Basic Medicine, Central South University, Changsha, 410000 Hunan China

**Biomarker Research (2024) 12:151**

10.1186/s40364-024-00693-8

The original article contained numerous minor typographical errors affecting subheading levels as well as an omission of a citation to Fig. 3, all of which have since been corrected.

